# Heavy Alcohol Exposure Activates Astroglial Hemichannels and Pannexons in the Hippocampus of Adolescent Rats: Effects on Neuroinflammation and Astrocyte Arborization

**DOI:** 10.3389/fncel.2018.00472

**Published:** 2018-12-04

**Authors:** Gonzalo I. Gómez, Romina V. Falcon, Carola J. Maturana, Valeria C. Labra, Nicole Salgado, Consuelo A. Rojas, Juan E. Oyarzun, Waldo Cerpa, Rodrigo A. Quintanilla, Juan A. Orellana

**Affiliations:** ^1^Departamento de Neurología, Escuela de Medicina and Centro Interdisciplinario de Neurociencias, Facultad de Medicina, Pontificia Universidad Católica de Chile, Santiago, Chile; ^2^Instituto de Ciencias Biomédicas, Facultad de Ciencias de la Salud, Universidad Autónoma de Chile, Santiago, Chile; ^3^Unidad de Microscopía Avanzada Medicina, Facultad de Medicina, Pontificia Universidad Católica de Chile, Santiago, Chile; ^4^Laboratorio de Función y Patología Neuronal, Departamento de Biología Celular y Molecular, Facultad de Ciencias Biológicas, Pontificia Universidad Católica de Chile, Santiago, Chile; ^5^Centro de Investigación y Estudio del Consumo de Alcohol en Adolescentes (CIAA), Santiago, Chile; ^6^Laboratory of Neurodegenerative Diseases, Universidad Autónoma de Chile, Santiago, Chile

**Keywords:** alcoholism, glia, connexins, pannexins, astrocyte, cytokines, hippocampus

## Abstract

A mounting body of evidence indicates that adolescents are specially more susceptible to alcohol influence than adults. However, the mechanisms underlying this phenomenon remain poorly understood. Astrocyte-mediated gliotransmission is crucial for hippocampal plasticity and recently, the opening of hemichannels and pannexons has been found to participate in both processes. Here, we evaluated whether adolescent rats exposed to ethanol exhibit changes in the activity of astrocyte hemichannels and pannexons in the hippocampus, as well as alterations in astrocyte arborization and cytokine levels. Adolescent rats were subjected to ethanol (3.0 g/kg) for two successive days at 48-h periods over 14 days. The opening of hemichannels and pannexons was examined in hippocampal slices by dye uptake, whereas hippocampal cytokine levels and astroglial arborization were determined by ELISA and Sholl analysis, respectively. We found that adolescent ethanol exposure increased the opening of connexin 43 (Cx43) hemichannels and pannexin-1 (Panx1) channels in astrocytes. Blockade of p38 mitogen-activated protein kinase (MAPK), inducible nitric oxide synthase (iNOS) and cyclooxygenases (COXs), as well as chelation of intracellular Ca^2+^, drastically reduced the ethanol-induced channel opening in astrocytes. Importantly, ethanol-induced Cx43 hemichannel and Panx1 channel activity was correlated with increased levels of interleukin-1β (IL-1β), tumor necrosis factor-α (TNF-α), IL-6 in the hippocampus, as well as with profound alterations in astrocyte arbor complexity. Thus, we propose that uncontrolled opening of astrocyte hemichannels and pannexons may contribute not only to the glial dysfunction and neurotoxicity caused by adolescent alcohol consumption, but also to the pathogenesis of alcohol use disorders in the adulthood.

## Introduction

Alcoholics exhibit a complex and multifactorial disorder featured as the repeated excessive use of alcoholic beverages, regardless of their prominent detrimental effects (Edenberg and Foroud, 2013). One pattern of alcohol misuse that has become popular among adolescents consist in consuming large amounts of alcohol in a single drinking session (~2 h), the latter with the intention of get intoxicated (Cservenka and Brumback, 2017). Heavy alcohol intake by young people often causes serious impairments in executive functions and memory, which correspond with structural changes in prefrontal and hippocampal brain regions (Brown et al., 2000; De Bellis et al., 2000; Bava and Tapert, 2010; Cohen-Gilbert et al., 2017). Because its gradual development during adolescence, the hippocampus seems to be especially vulnerable to immediate consequences of alcohol misuse, including blackouts, hangovers and alcohol poisoning (Zeigler et al., 2005). Heavy ethanol exposure impairs both spatial hippocampal-dependent memory and hippocampal long-term potentiation (LTP) in adolescent rats (Fernandes et al., 2018; Tapia-Rojas et al., 2018). Multiple hypotheses have been proposed to explain the ethanol-induced impairment in hippocampal synaptic plasticity, including glutamate and N-methyl-D-aspartate (NMDA) receptor dysfunction (Sabeti and Gruol, 2008), lower BDNF levels (Fernandes et al., 2018), decreased neurogenesis (Liu and Crews, 2017), increased cytokine levels (Cippitelli et al., 2017), DNA double-strand break (Suman et al., 2016) and mGlu1 receptor alterations (Reynolds et al., 2015), among others. Nevertheless, the effect of ethanol treatment in the interaction between astrocytes and neurons has not been studied in detail.

Astrocytes embody a wide-ranging syncytial network that anatomically and functionally establish dynamic and often bidirectional interactions with neuronal synapses (Gundersen et al., 2015). In companion with pre- and postsynaptic neuronal elements, astrocytes establish the “tripartite synapse,” a specialized functional platform in where they sense neurotransmission and respond to it by locally releasing paracrine substances called “gliotransmitters” (e.g., adenosine triphosphate (ATP), glutamate and D-serine; Araque et al., 1999; Perea et al., 2009). Astrocyte-mediated gliotransmission is crucial for hippocampal synaptic transmission and recently the opening of hemichannels and pannexons has been found to participate in this process (Ardiles et al., 2014; Chever et al., 2014; Meunier et al., 2017; Gajardo et al., 2018). Hemichannels are plasma membrane channels composed of six connexin subunits that oligomerize around a central pore that allow the passage of ions and small molecules, serving as a diffusional route of communication between the cytosol and the extracellular space (Montero and Orellana, 2015). In mammals, connexins exhibit an ubiquitous expression with 21 genes in humans and 20 in mice (Abascal and Zardoya, 2013). At the other end, pannexin channels or pannexons result from the oligomerization of six pannexins, a three-member family of proteins that have similar secondary and tertiary structures than connexins (Sosinsky et al., 2011). In astrocytes, hemichannels and pannexons allow the release of gliotransmitters that are necessary for different brain functions such as synaptic transmission and plasticity (Chever et al., 2014; Meunier et al., 2017), memory consolidation (Stehberg et al., 2012; Walrave et al., 2016), neuronal oscillations (Roux et al., 2015) and neuron-glia crosstalk (Torres et al., 2012). Despite the above, a mounting body of evidence have pointed out that homeostatic disturbances detected during brain diseases could be linked to enhanced and permanent opening of hemichannels and pannexons (Salameh et al., 2013; Orellana et al., 2014a, 2016).

Although several studies have demonstrated that alcohol exposure causes astrogliosis and alters the inflammatory profile of astrocytes (Blanco and Guerri, 2007; Adermark and Bowers, 2016), the molecular mechanisms and consequences of these phenomena are still poorly elucidated. Here, we show that intermittent ethanol exposure enhances the opening of connexin-43 (Cx43) hemichannels and pannexin-1 (Panx1) channels in hippocampal astrocytes from adolescent rats. In addition, we found that activation of (i) p38 mitogen-activated protein kinase (MAPK); (ii) inducible nitric oxide synthase (iNOS); (iii) cyclooxygenases (COXs); and (iv) cytoplasmic Ca^2+^; appear to be critical for the latter response. In agreement with the idea of inflammation as major cause of the ethanol-evoked hemichannel/pannexon activity, we also observed elevated levels of cytokines (interleukin-1β (IL-1β), tumor necrosis factor-α (TNF-α), IL-6) and altered arborization of astrocytes in the hippocampus of adolescent rat exposed to ethanol.

## Materials and Methods

### Reagents and Antibodies

The mimetic peptides gap19 (KQIEIKKFK, intracellular loop domain of Cx43), TAT-L2 (YGRKKRRQRRRDGANVDMHLKQIEIKKFKYGIEEHGK, second intracellular loop domain of Cx43) and ^10^panx1 (WRQAAFVDSY, first extracellular loop domain of Panx1) were obtained from GenScript (Piscataway Township, NJ, USA). Ethanol was obtained from Merck Millipore (Darmstadt, Germany). HEPES, ns-398, sc-560, L-N6, SB203580, lanthanum (La^3+^), anti-glial fibrillary acidic protein (GFAP) monoclonal antibody, BAPTA-AM, carbenoxolone (CBX), ethidium (Etd) bromide and probenecid were purchased from Sigma-Aldrich (St. Louis, MO, USA). Goat anti-mouse Alexa Fluor 488/555 and Hoechst 33342 were obtained from Thermo Fisher (Waltham, MA, USA). Normal goat serum (NGS) was purchased from Zymed (San Francisco, CA, USA).

### Animals

Male Sprague-Dawley rat pups, postnatal day 25 (PDN25), were housed in groups of three rats per cage and maintained to 22°C on 12:12 h light-dark cycle, with food and water *ad libitum* previous to heavy ethanol administration. The animals were treated and handled according to the National Institutes of Health guidelines (NIH, Baltimore, MD, USA). This study was carried out in accordance with the recommendations of the Animal Care Guidelines of the Research Ethic Committee from the Pontificia Universidad Católica de Chile. The protocol was approved by Research Ethic Committee from the Pontificia Universidad Católica de Chile.

### Protocol of Intermittent Ethanol Exposure

Doses of ethanol (3.0 g/kg, 25% w/v mixed in isotonic saline) or saline solution were administrated via intraperitoneal (i.p.) injections beginning on PND25 as previously described (Pascual et al., 2007). A second dose was given on PND26 followed by alternating injections in PND 29, 30, 33, 34, 37 and 38. The injected i.p. volumes were dependent on the weight of each animal. According to these variations in the time, amounts administered were 1–3 ml. As previously reported (Pascual et al., 2007), a single dose of ethanol using this protocol result in a maximum blood ethanol concentration (BEC) of 210 ± 11 mg/dL at 30 min post-injection, followed by a gradual decline at 540 min later.

### Enzyme-Linked Immunosorbent Assay (ELISA)

Enzyme-linked immunosorbent assay (ELISA) assays were performed to determine the amount of TNF-α, IL-1β and IL-6 in the hippocampus. Rats were anesthetized with ketamine/xylazine (10:1 mg/kg of body weight, i.p.) and then perfused and decapitated. Afterwards the hippocampus was removed and homogenized with an Ultra-Turrax homogenizer in buffer containing Tris-HCl 100 mM pH 7.4, EDTA 5 mM, SDS 1%, PMSF 1 μM and the protease inhibitor cocktail (ratio: 0.1 g hippocampus tissue: 1 ml lysis buffer; Pierce, Rockford, IL, USA). Protein concentrations were determined by using a detergent-compatible Bio-Rad protein assay kit (Bio-Rad, Richmond, CA, USA). Then, the samples were centrifuged at 14,000 *g* for 10 min. Supernatants were collected and protein content assayed by BCA method. Cytokines levels were determined by sandwich ELISA, according to the manufacturer’s protocol (IL-6, IL-1β and TNF-α EIA kit, Enzo Life Science, Farmingdale, NY, USA). For the assay, 100 μL of samples were added per ELISA plate well and incubated at 4°C overnight. A calibration curve with recombinant cytokine was included. Detection antibody was incubated at room temperature for 2 h and the reaction developed with avidin–HRP and substrate solution. Absorbance was measured at 450 nm with reference to 570 nm with the microplate reader Synergy HT (Biotek Instruments). The results were normalized by protein amount in ng/ml.

### Acute Brain Slices

Rats were anesthetized under isoflurane, decapitated and brains were extracted and cut into coronal slices (300 μm) using a vibratome (Leica, VT1000GS; Leica, Wetzlar, Germany) filled with ice-cold slicing solution containing (in mM): sucrose (222); KCl (2.6); NaHCO_3_ (27); NaHPO_4_ (1.5); glucose (10); MgSO_4_ (7); CaCl_2_ (0.5) and ascorbate (0.1), bubbled with 95% O_2_/5% CO_2_. Then, the slices were transferred at room temperature (20–22°C) to a holding chamber in ice-cold artificial cerebral spinal fluid (ACSF) containing (in mM): 125 NaCl, 2.5 KCl, 25 glucose, 25 NaHCO_3_, 1.25 NaH_2_PO_4_, 2 CaCl_2_ and 1 MgCl_2_, bubbled with 95% O_2_/5% CO_2_, pH 7.4, for a stabilization period of 60 min before dye uptake experiments (see below).

### Dye Uptake in Acute Brain Slices and Confocal Microscopy

For dye uptake and *ex vivo* “snapshot” experiments, acute brain slices were incubated with 5 μM Etd for 10 min in a chamber filled with ACSF and bubbled with 95% O_2_/5% CO_2_, pH 7.4. Some acute brain slices were pre-incubated for 15 min before and during Etd uptake experiments with the following agents: La^3+^ (200 μM), probenecid (500 μM), CBX (10 μM), gap19 (100 μM), TAT-L2 (100 μM), ^10^panx1 (100 μM); and ns-398 (10 μM), sc-560 (40 μM), SB203580 (10 μM); L-N6 (1 μM) and BAPTA (10 μM; Table 1). Afterwards, the slices were washed three times (5 min each) with ACSF, and fixed at room temperature with 4% paraformaldehyde for 60 min, rinsed once with 0.1 mM glycine in phosphate buffered saline (PBS) for 5 min and then twice with PBS for 10 min with gentle agitation. Then, the slices were incubated two times for 30 min each with a blocking solution (PBS, gelatin 0.2%, Triton-X 100 1%) at room temperature. Afterwards, the slices were incubated overnight at 4°C with anti-GFAP monoclonal antibody (1:500, Sigma) to detect astrocytes. Later, the slices were washed three times (10 min each) with blocking solution and then incubated for 2 h at room temperature with goat anti-mouse Alexa Fluor 488 (1:1,000) antibody and Hoechst 33342. Further, the slices were washed three times (10 min each) in PBS and then mounted in Fluoromount, cover-slipped and examined in a confocal laser-scanning microscope (TBCS SP2, Nikon, Japan). Stacks of consecutive confocal images were taken with 40× objective at 100 nm intervals were acquired sequentially with three lasers (in nm: 408, 488 and 543), and Z projections were reconstructed using Nikon confocal software (NIS-elements) and ImageJ software. Dye uptake was calculated with the following formula: Corrected total cell Etd fluorescence = Integrated Density − ([Area of selected cell] × [Mean fluorescence of background readings]). At least six cells per field were selected from at least three fields in each brain slice.

**Table 1:** Principal pharmacological agents used in this study.

Agent	Action
^10^panx1	Mimetic peptide against Panx1 channels
BAPTA-AM	Intracellular calcium chelator
CBX	General hemichannel and pannexon blocker
Gap19	Mimetic peptide against Cx43 hemichannels
La^3+^	General hemichannel blocker
L-N6	Selective inhibitor of iNOS
NS-398	Selective COX_2_ inhibitor
Probenecid	Panx1 hemichannel blocker
SB203580	p38 MAP Kinase Inhibitor
SC-560	Selective COX_1_ inhibitor
TAT-L2	Mimetic peptide against Cx43 hemichannels

### Quantification of Astrocyte Morphology and Sholl Analysis

Image processing was performed using the Fiji-ImageJ software (Schindelin et al., 2012). All samples were coded and analyzed randomly by a researcher blinded to animal number and condition. A minimum of 10 astrocytes from each animal where chosen for analysis and their image data were imported using the BioFormats plugin and then channels separated with the Split channels tool. Later, the GFAP channel was selected and Z-axis projection of the sum of planes was performed using the Z projection tool. Afterwards, astrocytes were selected and cut with the crop tool to facilitate their analysis when they fulfilled the following criteria: (i) presence of untruncated processes; (ii) consistent and strong GFAP staining along the entire arborization field; and (iii) relative isolation from neighboring astrocytes to avoid overlap. Afterwards, GFAP signal was segmented with the threshold tool and converted to binary mask before its skeletonization with the skeletonize tool. The latter tool allowed to obtain segment length and any possible bifurcation of the skeletonized image analyzed with the Fiji-ImageJ software. Then, maximum and total branch length of astroglial processes, as well as the number of branches were measured with the AnalizeSkeleton plugin of Fiji-ImageJ. Further, the plugin Sholl analysis of Fiji-ImageJ was used to place concentric circles around the cell starting from the soma and radiating outward at increasing radial increments of 5 μm (Sholl, 1953). Different parameters were measured including the number of intersections (points where the astrocytic processes cross concentric rings), area under the Sholl curve, the maximum number of intersections, the radius of highest count of intersections (maximum intersect. radius) and the sum of intersections divided by intersecting radii (mean of intersections).

### Statistical Analysis

For each data group, results were expressed as mean ± standard error (SEM); n refers to the number of independent experiments. Detailed statistical results were included in the figure legends. Statistical analyses were performed using GraphPad Prism (version 7, GraphPad Software, La Jolla, CA, USA). Normality and equal variances were assessed by the Shapiro-Wilk normality test and Brown-Forsythe test, respectively. Unless otherwise stated, data that passed these tests were analyzed by unpaired *t*-test in case of comparing two groups, whereas in case of multiple comparisons, data were analyzed by one or two-way analysis of variance (ANOVA) followed, in case of significance, by a Tukey’s *post hoc* test. When data were heteroscedastic as well as not normal and with unequal variances, we used Mann-Whitney test in case of comparing two groups, whereas in case of multiple comparisons data are analyzed by Kruskal-Wallis test followed, in case of significance, by Dunn’s *post hoc* test. A probability of *P* < 0.05 was considered statistically significant.

## Results

### Intermittent Heavy Ethanol Exposure Enhances Cx43 Hemichannel and Panx1 Channel Activity in Hippocampal Astrocytes From Acute Brain Slices of Adolescent Rats

In adolescent rodents, heavy ethanol exposure disrupts hippocampal-dependent synaptic plasticity and memory (Fernandes et al., 2018; Tapia-Rojas et al., 2018), as well as neuronal survival (Broadwater et al., 2014). Given that uncontrolled opening of astrocyte hemichannels and pannexons has been linked to synaptic impairment and neuronal loss (Orellana et al., 2011a,b; Abudara et al., 2015; Yi et al., 2016), we investigated whether intermittent heavy ethanol exposure affects the functional activity of these channels in the hippocampal CA1 region. To address this, we examined hemichannel and pannexon activity by measuring Etd uptake in acute brain slices from adolescent rats after different weeks following the last saline or ethanol injection. Etd enters to the cytoplasm of normal cells through plasma membrane channels with large pores, including hemichannels and pannexons. After its intercalation with base pairs of DNA and RNA, Etd becomes fluorescent, reflecting the activity of channels (Schalper et al., 2008; Sáez and Leybaert, 2014). Etd uptake by GFAP-positive astrocytes on acute brain slices was evaluated in “snapshot” experiments in the stratum oriens, stratum pyramidale and stratum radiatum of the hippocampal CA1 region.

**Figure 1:**
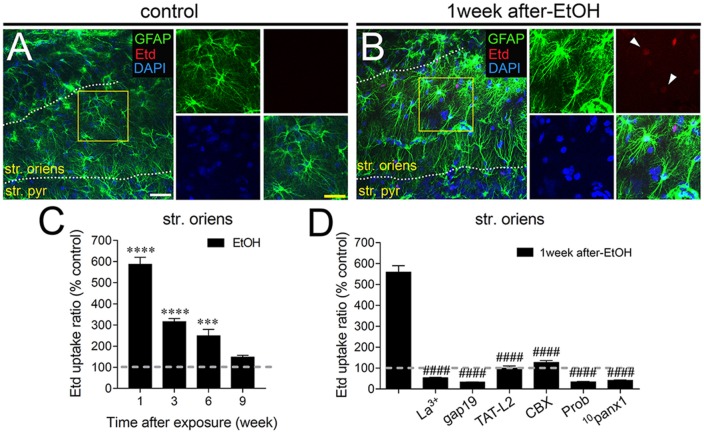
Intermittent heavy ethanol exposure enhances astroglial connexin 43 (Cx43) hemichannel and pannexin-1 (Panx1) channel activity in the stratum oriens of adolescent rats. Representative images showing glial fibrillary acidic protein (GFAP; green), ethidium (Etd; red) and DAPI (blue) staining in the stratum oriens of control rats **(A)** and rats after 1-week following ethanol exposure **(B)**. Insets of astrocytes were taken from the area depicted within the yellow squares in **(A,B)**. **(C)** Averaged data of Etd uptake normalized to control conditions (dashed line) by astrocytes in the stratum oriens from rats after different weeks following ethanol exposure. ****p < 0.0001, ***p = 0.0005, for the effect of post-ethanol exposure compared to the respective control condition (two-way analysis of variance (ANOVA) followed by Tukey’s *post hoc* test). Data were obtained from at least three independent experiments (≥59 cells analyzed for each independent experiment). **(D)** Averaged data of Etd uptake normalized to control conditions (dashed line) by astrocytes in the stratum oriens from rats after 1-week following ethanol exposure. Also shown are the effects of the following blockers applied during the Etd uptake assay: La^3+^ (200 μM), gap19 (100 μM), TAT-L2 (100 μM), CBX (5 μM), probenecid (Prob, 500 μM) and ^10^panx1 (100 μM). ^####^*p* < 0.0001, for the effect of 1-week post-ethanol exposure compared to the respective blocker (one-way ANOVA followed by Dunnett’s *post hoc* test). Data were obtained from at least three independent experiments (≥63 cells analyzed for each independent experiment). Calibration bars: white bar = 180 μm; yellow bar: 100 μm.

**Figure 2:**
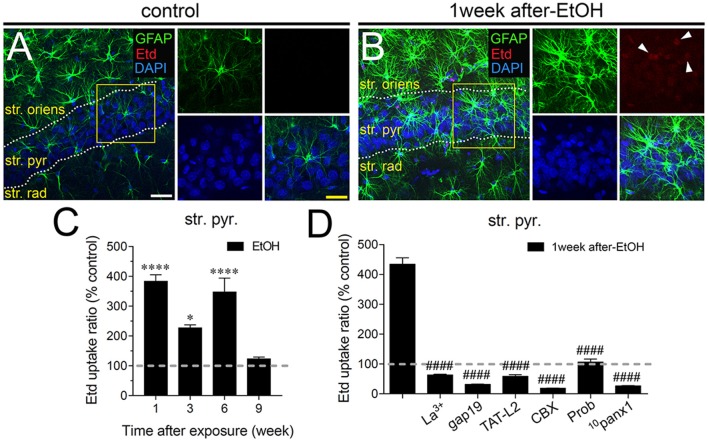
Intermittent heavy ethanol exposure enhances astroglial Cx43 hemichannel and Panx1 channel activity in the stratum pyramidale of adolescent rats. Representative images showing GFAP (green), Etd (red) and DAPI (blue) staining in the stratum oriens of control rats **(A)** and rats after 1-week following ethanol exposure **(B)**. Insets of astrocytes were taken from the area depicted within the yellow squares in **(A,B)**. **(C)** Averaged data of Etd uptake normalized to control conditions (dashed line) by astrocytes in the stratum oriens from rats after different weeks following ethanol exposure. ****p < 0.0001, *p = 0.0126 for the effect of post-ethanol exposure compared to the respective control condition (two-way ANOVA followed by Tukey’s *post hoc* test). Data were obtained from at least three independent experiments (≥58 cells analyzed for each independent experiment). **(D)** Averaged data of Etd uptake normalized to control conditions (dashed line) by astrocytes in the stratum oriens from rats after 1-week following ethanol exposure. Also shown are the effects of the following blockers applied during the Etd uptake assay: La^3+^ (200 μM), gap19 (100 μM), TAT-L2 (100 μM), carbenoxolone (CBX, 5 μM), probenecid (Prob, 500 μM) and ^10^panx1 (100 μM). ^####^*p* < 0.0001, for the effect of 1-week post-ethanol exposure compared to the respective blocker (one-way ANOVA followed by Dunnett’s *post hoc* test). Data were obtained from at least three independent experiments (≥55 cells analyzed for each independent experiment). Calibration bars: white bar = 180 μm; yellow bar: 100 μm.

**Figure 3:**
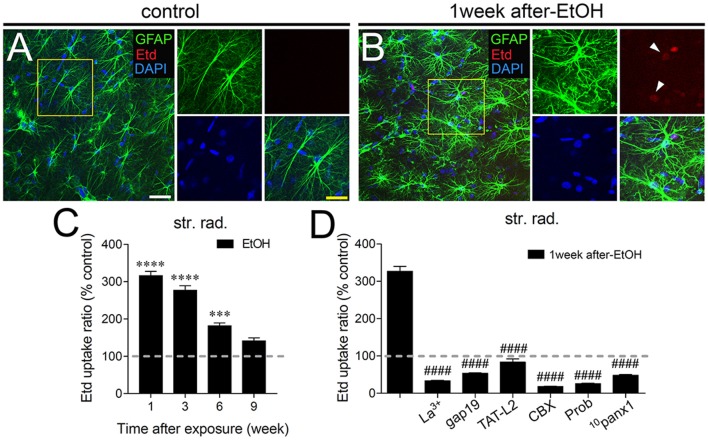
Intermittent heavy ethanol exposure enhances astroglial Cx43 hemichannel and Panx1 channel activity in the stratum radiatum of adolescent rats. Representative images showing GFAP (green), Etd (red) and DAPI (blue) staining in the stratum oriens of control rats **(A)** and rats after 1-week following ethanol exposure **(B)**. Insets of astrocytes were taken from the area depicted within the yellow squares in **(A,B)**. **(C)** Averaged data of Etd uptake normalized to control conditions (dashed line) by astrocytes in the stratum oriens from rats after different weeks following ethanol exposure. ****p < 0.0001, ***p = 0.0002 for the effect of post-ethanol exposure compared to the respective control condition (two-way ANOVA followed by Tukey’s *post hoc* test). Data were obtained from at least three independent experiments (≥59 cells analyzed for each independent experiment). **(D)** Averaged data of Etd uptake normalized to control conditions (dashed line) by astrocytes in the stratum oriens from rats after 1-week following ethanol exposure. Also shown are the effects of the following blockers applied during the Etd uptake assay: La^3+^ (200 μM), gap19 (100 μM), TAT-L2 (100 μM), CBX (5 μM), probenecid (Prob, 500 μM) and ^10^panx1 (100 μM). ^####^*p* < 0.0001, for the effect of 1-week post-ethanol exposure compared to the respective blocker (one-way ANOVA followed by Dunnett’s *post hoc* test). Data were obtained from at least three independent experiments (≥52 cells analyzed for each independent experiment). Calibration bars: white bar = 180 μm; yellow bar: 100 μm.

Astrocytes observed in acute brain slices from control adolescent rats exhibited a low Etd uptake ratio in all CA1 regions analyzed (Figures 1A, 2A, 3A). However, during the 1-week period after the last ethanol injection, adolescent rats showed astrocytes with increased Etd uptake at the stratum oriens (~560%, Figures 1A–C), stratum pyramidale (~430%, Figures 2A–C) and stratum radiatum (~325%, Figures 3A–C). Temporal analysis of these responses in acute brain slices revealed that astroglial Etd uptake rapidly augmented 1-week post ethanol exposure but progressively decreased as the weeks following ethanol injections increased (Figures 1C, 2C, 3C). Given that Cx43 hemichannels and Panx1 channels are major routes for dye influx in astrocytes (Contreras et al., 2002; Iglesias et al., 2009), the possible role of these channels in the ethanol-induced astroglial Etd uptake was examined. To do that, some acute brain slices were pre-incubated for 15 min before and during Etd uptake experiments with different pharmacological agents (Table 1). La^3+^ (200 μM), a general hemichannel blocker (Schalper et al., 2008), or the two Cx43 hemichannel mimetic peptides gap19 (100 μM) and TAT-L2 (100 μM; Wang et al., 2013), strongly reduced the astroglial Etd uptake observed in the stratum oriens after 1 week following ethanol exposure (from ~560% to ~52%, ~33% and ~100%, respectively; Figure 1D). These inhibitors caused similar counteracting actions in the ethanol-mediated Etd uptake detected in astrocytes from the stratum pyramidale (Figure 2D) and stratum radiatum (Figure 3D). To figure out the implication of Panx1 channels in the astroglial Etd uptake observed in acute brain slices of ethanol-exposed rats, we employed CBX and probenecid, two well-known blockers of these channels (Silverman et al., 2008; D’Hondt et al., 2009); as well as the Panx1 mimetic peptide: ^10^panx1 (Pelegrin and Surprenant, 2006). CBX (5 μM), probenecid (500 μM) and ^10^panx1 (100 μM) strongly blunted the astroglial Etd uptake induced by 1-week post-ethanol exposure in the stratus oriens (Figure 1D), stratum pyramidale (Figure 2D) and stratum radiatum (Figure 3D). Altogether, these data robustly denote that intermittent ethanol exposure increases the activity of Cx43 hemichannels and Panx1 channels in astrocytes from different hippocampal CA1 regions of adolescent rats.

### Astrocyte Cx43 Hemichannel and Panx1 Channel Activity Mediated by Intermittent Heavy Ethanol Exposure Depends on p38 MAP Kinase/iNOS/COXs Pathways

**Figure 4:**
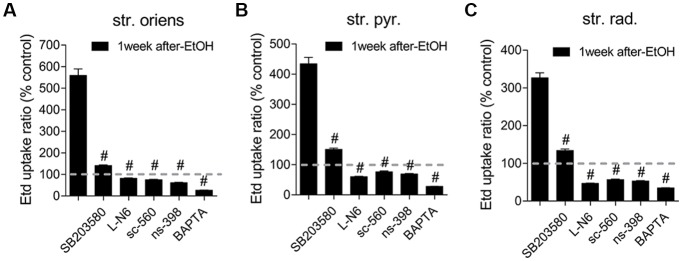
Hemichannel and pannexon activity induced by intermittent heavy ethanol exposure depends on p38 mitogen-activated protein kinase (MAPK)/inducible nitric oxide synthase (iNOS)/cyclooxygenases (COXs) pathways. **(A–C)** Averaged data of Etd uptake normalized to control conditions (dashed line) by astrocytes in the stratum oriens **(A)**, stratum pyramidale **(B)** and stratum radiatum **(C)** from rats after 1-week following ethanol exposure. Also shown are the effects of the following blockers applied during the Etd uptake assay: SB203580 (10 μM, p38 MAPK inhibitor), LN-6 (1 μM, iNOS inhibitor), sc-560 (40 μM, COX_1_ inhibitor), ns-398 (10 μM, COX2 inhibitor) and BAPTA (10 μM, Ca^2+^ chelator). ^#^*p* < 0.0001, for the effect of 1-week post-ethanol exposure compared to the respective blocker (one-way ANOVA followed by Dunnett’s *post hoc* test). Data were obtained from at least three independent experiments (≥90 cells analyzed for each independent experiment).

In astrocytes, *in vivo* or *in vitro* ethanol exposure causes activation of iNOS and COX_2_; (Blanco et al., 2004; Davis and Syapin, 2004; Vallés et al., 2004). Both enzymes generate NO and prostaglandins (PGs), respectively, two byproducts linked to the opening of astroglial Cx43 hemichannels (Retamal et al., 2006; Orellana et al., 2014b; Avendaño et al., 2015). Previous studies have revealed the involvement of p38 MAPK in the activity of Cx43 hemichannels (Retamal et al., 2006) and inflammatory activation of astrocytes (Pascual et al., 2003; Vallés et al., 2004; Blanco et al., 2005). Accordingly, we examined the influence of p38 MAPK, iNOS, COX_1_ and COX_2_ activation in the astrocyte Etd uptake observed in acute brain slices of ethanol-exposed adolescent rats. The Etd uptake triggered during the 1-week period following the last ethanol injection was strongly reduced by inhibition of p38 MAPK with 10 μM SB202190 or blockade of iNOS with 1 μM L-N6 in the three CA1 regions analyzed: stratus oriens (to ~140% and ~82%, respectively; Figure 4A), stratum pyramidale (to ~150% and ~60%, respectively; Figure 4B) and stratum radiatum (to ~134% and ~47%, respectively; Figure 4C). Likewise, sc-560 (40 μM) and ns-398 (10 μM), specific inhibitors for COX_1_ and COX_2_, respectively, prominently neutralized the Etd uptake caused by 1-week post-ethanol exposure in astrocytes from stratum oriens, stratum pyramidale and stratum radiatum (Figures 4A–C).

Prior evidence indicates that NO enhances COX_2_ function and PG E_2_ (PGE_2_) production in macrophages (Swierkosz et al., 1995) and a similar phenomenon seems to occur in astrocytes treated with ethanol (Pascual et al., 2003; Blanco et al., 2005). Because activation of PGE_2_ receptor 1 (EP_1_) raises [Ca^2+^]_i_ levels (Woodward et al., 2011) and the latter is a broad-recognized cascade that elevates the opening of Cx43 hemichannels (De Bock et al., 2012) and Panx1 channels (Locovei et al., 2006), we examined whether [Ca^2+^]_i_ is involved in astrocyte Etd uptake observed in the hippocampus of ethanol-exposed rats. Treatment with 5 μM BAPTA, a Ca^2+^ chelator, was found to reduce the Etd uptake caused by 1-week post-ethanol exposure in stratus oriens (to ~26%, Figure 4A), stratum pyramidale (to ~28%, Figure 4B) and stratum radiatum (to ~35%, Figure 4C). All these data suggest that Cx43 hemichannel and Panx1 channel opening in our system depends on the activation of p38 MAPK/iNOS/COXs–mediated pathway(s) and changes in cytoplasmic Ca^2+^ signaling.

### Intermittent Heavy Ethanol Exposure Increases Hippocampal Cytokine Levels and Alters Astrocyte Arborization in Adolescent Rats

**Figure 5:**
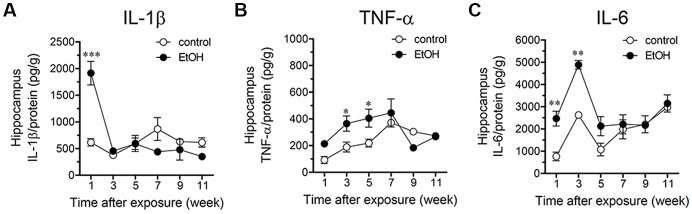
Intermittent heavy ethanol exposure increases hippocampal levels of interleukin-1β (IL-1β), tumor necrosis factor-α (TNF-α) and IL-6. Averaged data of hippocampal levels of IL-1β **(A)**, TNF-α **(B)** and IL-6 **(C)** from control rats (white circles) or rats after different weeks following ethanol exposure (black circles). *p < 0.05; **p < 0.0005, ***p < 0.0001; effect of ethanol exposure compared with control conditions (two-way ANOVA followed by Bonferroni’s *post hoc* test). Averaged data were obtained from at least three animals.

Once activated, astrocytes release relevant autocrine/paracrine amounts of inflammatory cytokines, which are capable of modify astrocyte properties at the molecular, morphological and functional level (Rossi and Volterra, 2009; Agulhon et al., 2012). Previous studies have shown that different cytokines such as IL-1β, IFN-γ, IL-6 and TNF-α elevate the opening of hemichannels and pannexons in different cell types (Takeuchi et al., 2006; Sáez et al., 2013; Mugisho et al., 2018), including astrocytes (Retamal et al., 2007; Froger et al., 2010). More relevantly, both hemichannels and pannexons have been proposed to contribute to the stimulation of the inflammasome route and its propagation through the release of cytokines to neighboring cells (Makarenkova and Shestopalov, 2014; Kim et al., 2016). Multiple lines of evidence have demonstrated that ethanol may increase the release of IL-1β, TNF-α and IL-6 from astrocytes through the activation of p38 MAPK/iNOS/COXs pathway(s). Given that inhibition of the latter pathways significantly reduced the astrocyte hemichannel/pannexon activity in ethanol-exposed rats, we evaluated whether ethanol could affect the levels of IL-1β, IFN-γ and IL-6 in the hippocampus. During the 1-week period after the last ethanol injection, the hippocampus of ethanol-exposed rats exhibited a prominent ~5-fold increase in IL-1β levels that then dropped drastically during the following weeks (Figure 5A). Similarly, following 3-week of the last injection, ethanol caused a significant 2-fold steady-state increase in hippocampal TNF-α levels that was maintained for 2 weeks after returning to control levels (Figure 5B). The hippocampus of rats injected with ethanol also exhibited a 2.5-fold increase in IL-6 levels during 3-weeks following ethanol exposure, which did not persist further (Figure 5C). Collectively, these results indicate that intermittent ethanol injections cause a rapid and transient inflammation in the hippocampus of adolescent rats.

**Figure 6:**
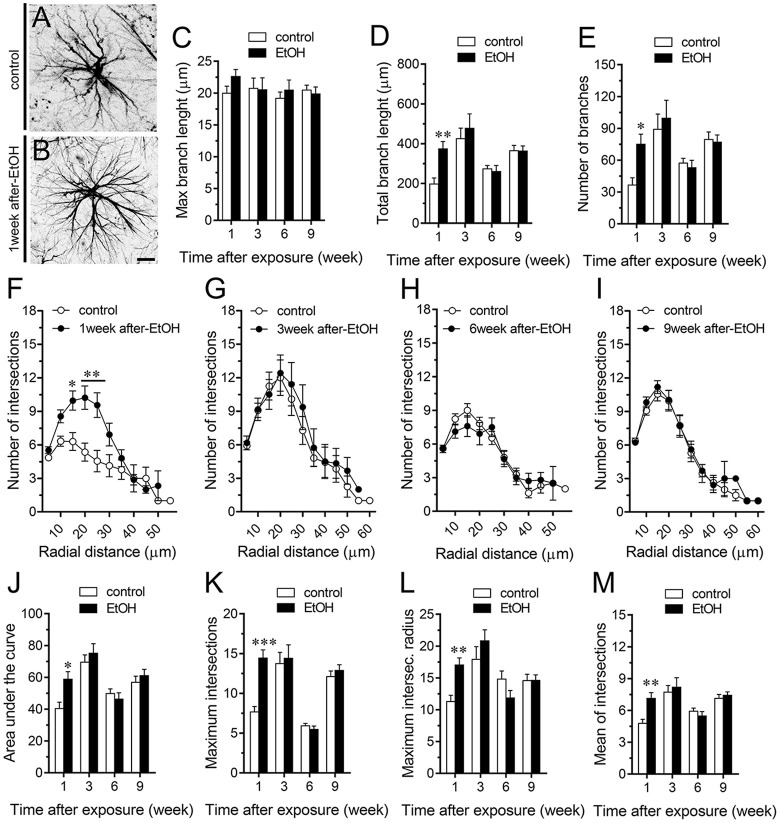
Intermittent heavy ethanol exposure increases astrocyte arborization in the stratum oriens of adolescent rats.** (A,B)** Representative GFAP (black) positive astrocytes from the stratum oriens of control rats **(A)** and rats after 1-week following ethanol exposure **(B)**. **(C–E)** Averaged data of maximum branch length **(C)**, total branch length **(D)** and number of branches **(E)** by astrocytes in the stratum oriens from control rats (white bars) or rats after different weeks following ethanol exposure (black bars). **p < 0.0005, *p < 0.002, for the effect of post-ethanol exposure compared to the respective control condition (two-way ANOVA followed by Bonferroni’s *post hoc* test). **(F–I)** Averaged data of Sholl analysis astrocytes from the stratum oriens of control rats (white circles) and rats after 1 **(F)**, 3 **(G)**, 6 **(H)** and 9 **(I)** weeks following ethanol exposure (black circles). *p < 0.005, **p < 0.0001 for the effect of post-ethanol exposure compared to the respective control condition (two-way ANOVA followed by Bonferroni’s *post hoc* test). **(J–M)** Averaged data of area under the curve of Sholl analysis **(J)**, maximum intersection **(K)**, maximum intersection radius **(L)** and mean of intersections **(M)** by astrocytes in the stratum oriens from control rats (white bars) or rats after different weeks following ethanol exposure (black bars). ***p < 0.0001, **p < 0.005, *p < 0.05, for the effect of post-ethanol exposure compared to the respective control condition (two-way ANOVA followed by Bonferroni’s *post hoc* test). Data were obtained from at least three independent experiments. Calibration bar = 25 μm.

**Figure 7:**
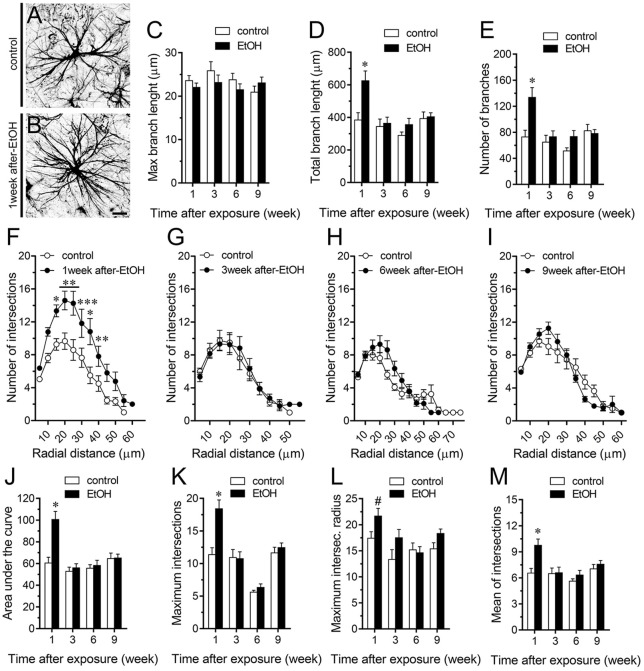
Intermittent heavy ethanol exposure increases astrocyte arborization in the stratum pyramidale of adolescent rats.** (A,B)** Representative GFAP (black) positive astrocytes from the stratum pyramidale of control rats **(A)** and rats after 1-week following ethanol exposure **(B)**. **(C–E)** Averaged data of maximum branch length **(C)**, total branch length **(D)** and number of branches **(E)** by astrocytes in the stratum pyramidale from control rats (white bars) or rats after different weeks following ethanol exposure (black bars). *p < 0.0001, for the effect of post-ethanol exposure compared to the respective control condition (two-way ANOVA followed by Bonferroni’s *post hoc* test). **(F–I)** Averaged data of Sholl analysis astrocytes from the stratum pyramidale of control rats (white circles) and rats after 1 **(F)**, 3 **(G)**, 6 **(H)** and 9 **(I)** weeks following ethanol exposure (black circles). ***p < 0.0006, **p < 0.005, *p < 0.05, for the effect of post-ethanol exposure compared to the respective control condition (two-way ANOVA followed by Bonferroni’s *post hoc* test). **(J–M)** Averaged data of area under the curve of Sholl analysis **(J)**, maximum intersection **(K)**, maximum intersection radius **(L)** and mean of intersections **(M)** by astrocytes in the stratum pyramidale from control rats (white bars) or rats after different weeks following ethanol exposure (black bars). *p < 0.0001, for the effect of post-ethanol exposure compared to the respective control condition (two-way ANOVA followed by Bonferroni’s *post hoc* test). ^#^*p* < 0.05, for the effect of post-ethanol exposure compared to the respective control condition (Mann Whitney test). Data were obtained from at least three independent experiments. Calibration bar = 25 μm.

**Figure 8:**
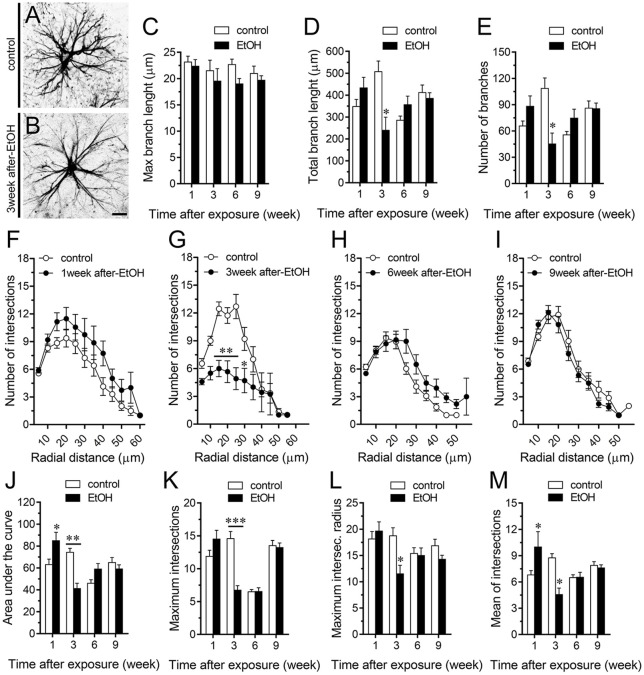
Intermittent heavy ethanol exposure modulates astrocyte arborization in the stratum radiatum of adolescent rats. **(A,B)** Representative GFAP (black) positive astrocytes from the stratum radiatum of control rats **(A)** and rats after 3-week following ethanol exposure **(B)**. **(C–E)** Averaged data of maximum branch length **(C)**, total branch length **(D)** and number of branches **(E)** by astrocytes in the stratum radiatum from control rats (white bars) or rats after different weeks following ethanol exposure (black bars). *p < 0.002, for the effect of post-ethanol exposure compared to the respective control condition (two-way ANOVA followed by Bonferroni’s *post hoc* test). **(F–I)** Averaged data of Sholl analysis astrocytes from the stratum radiatum of control rats (white circles) and rats after 1 **(F)**, 3 **(G)**, 6 **(H)** and 9 **(I)** weeks following ethanol exposure (black circles). **p < 0.0002, *p < 0.05, for the effect of post-ethanol exposure compared to the respective control condition (two-way ANOVA followed by Bonferroni’s *post hoc* test). **(J–M)** Averaged data of area under the curve of Sholl analysis **(J)**, maximum intersection **(K)**, maximum intersection radius **(L)** and mean of intersections **(M)** by astrocytes in the stratum radiatum from control rats (white bars) or rats after different weeks following ethanol exposure (black bars). ***p < 0.0001, **p < 0.001, *p < 0.05, for the effect of post-ethanol exposure compared to the respective control condition (two-way ANOVA followed by Bonferroni’s *post hoc* test). Data were obtained from at least three independent experiments. Calibration bar = 25 μm.

A large number of studies have shown that inflammation evoked by ethanol exposure is accompanied of reactive astrogliosis (Blanco and Guerri, 2007; Adermark and Bowers, 2016). The latter is a multiple stage and preserved process by which astrocytes undergo molecular, morphological and functional changes to counteract and limit brain damage (Pekny and Pekna, 2014). Hallmark features of reactive astrogliosis are the hypertrophy of cellular processes and profound alterations in the arborization and morphology of astrocytes (Pekny and Pekna, 2014). To examine whether ethanol exposure could affect the extent of astroglial arbor in the hippocampus, we measured the maximum and total branch length of astrocytes at the stratum oriens (Figures 6A–D), stratum pyramidale (Figures 7A–D) and stratum radiatum (Figures 8A–D). Measures beginning in the cell body throughout the end of each process, allowed us to calculate the length of the longest branch and the sum of all branch lengths of each astrocyte arbor, which were represented as maximum and total branch length, respectively. Temporal analysis of astrocyte arbor at the hippocampal CA1 area showed that maximum branch length remained unchanged between control rats and rats following different periods after ethanol exposure (Figures 6C, 7C, 8C). However, 1-week post-ethanol exposure strongly increased the total branch length of astrocytes in the stratum oriens (~2-fold, Figure 6D) and stratum pyramidale (~1.5-fold, Figure 7D) of adolescent rats. In an intriguing contrast, during the 3-week period after the last ethanol injection, adolescent rats exhibited astrocytes with a 2-fold reduction in total branch length in the stratum radiatum as compared to the control ones (Figure 8D). Similarly, 1-week post-ethanol exposure greatly elevated the number of astrocyte branches in the stratum oriens (~2-fold, Figure 6E) and stratum pyramidale (~2-fold, Figure 7E), nevertheless, an opposite response was observed in the stratum radiatum during the 3-week period after the last ethanol injection (Figure 8E).

To further scrutinize with major detail the arbor complexity of astrocytes in brain sections from control and ethanol-exposed rats, we used a Sholl analysis, which consist in place concentric rings at fixed intervals from the soma to further count branch intersections at each ring. Based on Sholl analysis, hippocampal astrocytes of ethanol-exposed rats are significantly different from hippocampal astrocytes of their saline-injected counterparts (Figures 6F–M, 7F–M, 8F–M). We found an increased number of intersections between branches and Sholl rings in ethanol-exposed rats, particularly in the stratum oriens and stratum pyramidale after 1-week but not in further periods following ethanol injections (Figures 6F–I, 7F–I). In these hippocampal areas, 1-week post-ethanol exposure also augmented astrocyte branch complexity as assessed by the area under the Sholl curve for the total number of branch intersections at 5–60 μm from the soma (Figures 6J, 7J). Ethanol exposure evoked similar increased values in the maximum number of intersections, the radius of highest count of intersections (maximum intersect. radius) and the sum of intersections divided by intersecting radii (mean of intersections; Figures 6K–M, 7K–M). Although 1-week post-ethanol exposure significantly elevated the area under the Sholl curve and mean intersections in the stratum radiatum, a 2-fold decrease in all Sholl parameters was observed after 3-week following ethanol injections in this region (Figures 8D–M). These data indicate that ethanol modulates the complexity of astrocyte branch arbors in the hippocampus of adolescent rats in a time and spatial-depend manner.

## Discussion

In the present study, we showed that intermittent heavy ethanol exposure enhances the opening of Cx43 hemichannels and Panx1 channels in hippocampal astrocytes from acute brain slices of adolescent rats. This enhanced channel activity took place by a mechanism involving the stimulation of p38 MAP k/iNOS/COX-dependent pathways and intracellular Ca^2+^ signaling. The above responses were accompanied of elevated levels of IL-1β, TNF-α, IL-6 and altered arborization of astrocytes in the hippocampus. All these effects persisted during early adulthood but then were progressively compensated overtime.

Prior evidence indicates that heavy ethanol exposure during adolescence disturbs spatial hippocampus-mediated cognitive ability and LTP (Fernandes et al., 2018; Tapia-Rojas et al., 2018), as well as neuronal survival (Broadwater et al., 2014). Our study suggests that a portion of the above-mentioned alterations caused by ethanol exposure could base in the persistent opening of Cx43 hemichannels and Panx1 channels within the hippocampus. During the past decade, different lines of research have established that hemichannels and pannexons underpin multiple brain processes such as synaptic efficacy, neural activity, signal processing, cognition and behavior (Stehberg et al., 2012; Ardiles et al., 2014; Chever et al., 2014; Roux et al., 2015; Walrave et al., 2016; Meunier et al., 2017; Gajardo et al., 2018). However, during certain pathophysiological conditions, hemichannels and pannexons may favor brain disease progression by: (i) releasing excitotoxic levels of transmitters (e.g., ATP and glutamate); (ii) disturbing [Ca^2+^]_i_ handling; or (iii) altering cytoplasmic ionic and osmotic balance (Vicario et al., 2017). Here, we found that intermittent ethanol injections during adolescence increase the opening of Cx43 hemichannels and Panx1 channels in hippocampal astrocytes from the stratum oriens, stratum pyramidale and stratum radiatum. These responses were drastically blocked by La^3+^, gap19, TAT-L2, CBX, probenecid and ^10^panx1, indicating that both Cx43 hemichannels and Panx1 channels were the principal responsible for the ethanol-mediated Etd uptake in astrocytes. The latter coincides with the increased activity described for both channels in astrocytes during several brain pathological scenarios including prenatal nicotine and postnatal high-fat diet (Orellana et al., 2014b), restraint stress (Orellana et al., 2015), epileptic seizures (Santiago et al., 2011), amyloid-β peptide treatment (Orellana et al., 2011b), spinal cord injury (Garré et al., 2016) and acute infection (Karpuk et al., 2011).

**Figure 9:**
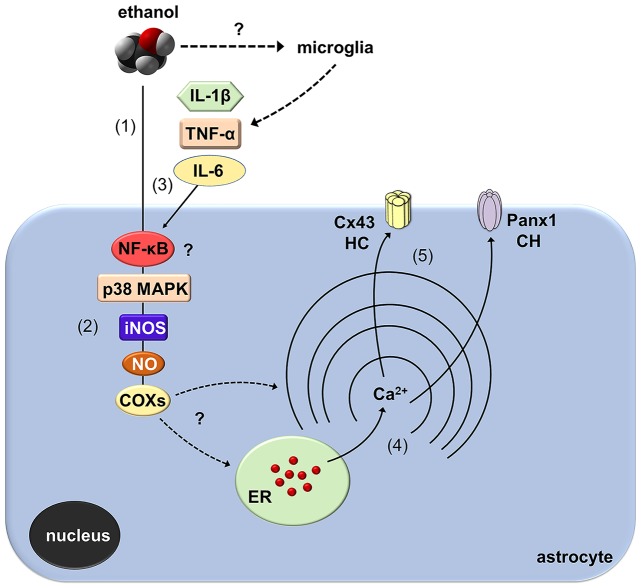
Schematic diagram showing the possible pathways involved in the activation of astroglial Cx43 hemichannels and Panx1 channels evoked by *in vivo* ethanol exposure during adolescence. Ethanol could stimulate directly astrocytes (1) with a signal transduction that possibly involves NF-κβ signaling associated to p38 MAPK and iNOS activation, NO production and further stimulation of COXs (2). Alternatively, activation of NF-κβ signaling in astrocytes and/or microglia could evoke the autocrine/paracrine release of IL-1β, TNF-α, and IL-6, which are well-known inducers of the opening of hemichannel (HC) and pannexons via the stimulation of p38 MAPK/iNOS/COX pathway (3). The latter cascade through an unknown mechanism likely increase cytoplasmic levels of Ca^2+^ (4), which is known to open Cx43 HCs and Panx1 channels (CHs) (5).

How does ethanol exposure during adolescence causes the opening of Cx43 hemichannels and Panx1 channels? Recent evidence indicates that hemichannel and pannexon activity in astrocytes result from the activation of a p38MAPK/iNOS/COX-dependent pathway (Retamal et al., 2006; Orellana et al., 2014b; Avendaño et al., 2015). Specifically, pro-inflammatory conditions (e.g., IL-1β and TNF-α) enhance the opening of Cx43 hemichannels via the stimulation of p38 MAPK and further NO-dependent S-nitrosylation of Cx43 (Retamal et al., 2006, 2007; Avendaño et al., 2015). Furthermore, COX_1_/COX_2_ stimulation is necessary for the persistent opening of Cx43 hemichannels and Panx1 channels elicited by prenatal nicotine and high-fat diet in the hippocampus (Orellana et al., 2014b). Similarly, iNOS and COX_2_ activity, as well as [Ca^2+^]_i_ and NO, base the Panx1 channel-mediated release of ATP in LPS-treated microglia (Orellana et al., 2013). Here, we demonstrated that ethanol-mediated Etd uptake in hippocampal astrocytes is totally suppressed by blockers of p38MAPK, iNOS, COX_1_ and COX_2_, implicating that activation of Cx43 and Panx1 unopposed channels possibly took place downstream of these pathways (Figure 9). Importantly, the activity of these channels also depended on [Ca^2+^]_i_ signaling as BAPTA completely abolished the Etd uptake evoked by ethanol. The above findings are consistent with three facts observed in previous studies: (i) ethanol induces the activation of iNOS and COXs in astrocytes (Pascual et al., 2003; Blanco et al., 2005); (ii) iNOS and COX activity raises [Ca^2+^]_i_ due the release of PGE_2_ and the subsequent stimulation of EP_1_ receptors (Swierkosz et al., 1995; Woodward et al., 2011), which are expressed in astrocytes (Fiebich et al., 2001); and (iii) the opening of hemichannels and pannexons rely on [Ca^2+^]_i_ increments (Locovei et al., 2006; De Bock et al., 2012; Meunier et al., 2017). Whether ethanol-induced hemichannel/pannexon activity occurs by parallel mechanisms affecting astrocytes, including post-translational modifications (e.g., S-nitrosylation) and [Ca^2+^]_i_ signaling, will be matter of future investigations. In addition, we cannot rule out the influence of microglia in the activity of Cx43 hemichannels and Panx1 channels observed in astrocytes from ethanol-exposed rats. Indeed, microglia are particularly sensitive to ethanol (Fernandez-Lizarbe et al., 2009) and also express functional Cx43 hemichannels and Panx1 channels (Gajardo-Gómez et al., 2016).

A keystone underlying the opening of hemichannels and pannexons in the nervous system came from the long-lasting production of pro-inflammatory cytokines due to the disrupted function of the brain innate and adaptive immune system (Sarrouilhe et al., 2017). Previous studies have demonstrated that several models of adolescent alcohol exposure raise cytokine levels in different brain areas (Pascual et al., 2018), including the hippocampus (Kane et al., 2014; Doremus-Fitzwater et al., 2015). Here, we observed that intermittent ethanol injections during adolescence cause rapid and transient augments in hippocampal levels of IL-1β, TNF-α and IL-6. Consequently, it is plausible to hypothesize that by activating the above signaling, IL-1β, TNF-α and IL-6 may be in part responsible of the opening of astroglial hemichannels and pannexons observed in our model of adolescent ethanol exposure (Figure 9). Supporting this idea, in astrocytes, the opening of Cx43 hemichannels and Panx1 channels is stimulated by IL-1β and TNF-α (Retamal et al., 2007; Avendaño et al., 2015), the latter cytokines being closely linked to the activation of p38 MAPK, iNOS and COXs (Clerk et al., 1999; Vinukonda et al., 2010; Sheng et al., 2011; Samy and Igwe, 2012). Alternatively, hemichannel and pannexon activity may result from alterations in redox status. During pathological scenarios, oxidant molecules increase the activity of Cx43 hemichannels and Panx1 channels (Retamal, 2014). In a recent work, we showed that the model of heavy ethanol exposure used in this study increases rapidly oxidative stress and mitochondrial dysfunction in the hippocampus (Tapia-Rojas et al., 2018). Whether astroglial hemichannel and pannexon opening evoked by ethanol exposure take places by an impairment in redox status and production of free radicals remains to be elucidated.

During neuroinflammation, among other changes, astrocytes undergo the remodeling of their dendritic arbor as well as multiple morphological alterations (Pekny and Nilsson, 2005). There is plenty of data demonstrating the detrimental effects of ethanol on astrocyte functions (Adermark and Bowers, 2016), but whether this occur in the adolescent brain is just beginning to be understood (Pascual et al., 2018). In the present study, we found that intermittent ethanol exposure modulates the complexity of astrocyte branch arbors in the hippocampus of adolescent rats in a time and spatial-depend manner. Particularly, ethanol exposure increased in a rapid and transient manner the arbor complexity of astrocytes in the stratum oriens and stratum pyramidale, but decreased branch arborization in the stratum radiatum after 3 weeks following ethanol exposure. These findings harmonize with previous studies showing the layer-specific arborization of astrocytes (Lanjakornsiripan et al., 2018) and the opposite regulation of this astroglial feature during different pathological conditions (Lechuga-Sancho et al., 2006; Torres-Platas et al., 2011; Tsai et al., 2018). Furthermore, in the hippocampus, astrocytes show a wide diversity and heterogeneity, particularly, astrocytes close to the stratum pyramidale are organized in networks that remain parallel to this layer, whereas astrocytes in the stratum radiatum have circular networks (Houades et al., 2006). Likewise, the electrophysiological features of hippocampal astrocytes also vary according to their location in different subregions of the hippocampus (Ben Haim and Rowitch, 2017). For example, astrocytes from the CA1 and CA3 areas of the hippocampus show different levels of cell-cell coupling (D’Ambrosio et al., 1998). This analysis becomes even more complex when the diversity of neuronal populations (e.g., pyramidal, baskets, etc.) and their projections (e.g., inhibitory, excitatory) are considered. These differences may explain not just the basal and ethanol-mediated heterogeneity in hippocampal astrocyte arborization, but also the diversity in ethanol-induced channel responses in the regions analyzed: stratum oriens > stratum pyramidale > stratum radiatum. Future studies are necessary to uncover the molecular and cellular mechanisms of these differential responses.

The fact that channel activity, cytokine production and astrocyte arborization were recovered to control levels during 3–7 weeks post-ethanol exposure is relevant and reveal the plasticity of astrocyte function and neural circuits. Our findings indicate that opening of hemichannels and pannexons occurs at early phases post alcohol exposure and is accompanied of neuroinflammation and astrocyte activation. We speculate that uncontrolled opening of astrocyte hemichannels and pannexons may contributes to the neurotoxicity caused by adolescent alcohol consumption. Future studies will elucidate whether these channels may participate in the pathogenesis of alcohol use disorders in the adulthood.

## Author Contributions

GG, RF, CM, VL, NS, CR, JEO, WC, RQ and JAO: conceived, performed and analyzed the experiments. JAO: wrote and edited the manuscript. All authors read and approved the final manuscript.

## Conflict of Interest Statement

The authors declare that the research was conducted in the absence of any commercial or financial relationships that could be construed as a potential conflict of interest.
